# Evolutionary patterns of nucleotide substitution rates in plastid genomes of *Quercus*

**DOI:** 10.1002/ece3.8063

**Published:** 2021-08-30

**Authors:** Xuan Li, Yongfu Li, Steven Paul Sylvester, Mingyue Zang, Yousry A. El‐Kassaby, Yanming Fang

**Affiliations:** ^1^ Key Laboratory of State Forestry and Grassland Administration on Subtropical Forest Biodiversity Conservation College of Biology and the Environment Co‐Innovation Center for Sustainable Forestry in Southern China Nanjing Forestry University Nanjing China; ^2^ Department of Forest and Conservation Sciences Faculty of Forestry The University of British Columbia Vancouver BC Canada

**Keywords:** chloroplast genome, evolutionary rate, nonsynonymous substitution, oaks, selective pressure, synonymous substitution

## Abstract

Molecular evolution, including nucleotide substitutions, plays an important role in understanding the dynamics and mechanisms of species evolution. Here, we sequenced whole plastid genomes (plastomes) of *Quercus fabri*, *Quercus semecarpifolia*, *Quercus engleriana*, and *Quercus phellos* and compared them with 14 other *Quercus* plastomes to explore their evolutionary relationships using 67 shared protein‐coding sequences. While many previously identified evolutionary relationships were found, our findings do not support previous research which retrieve *Quercus* subg. *Cerris* sect. *Ilex* as a monophyletic group, with sect. *Ilex* found to be polyphyletic and composed of three strongly supported lineages inserted between sections *Cerris* and *Cyclobalanposis*. Compared with gymnosperms, *Quercus* plastomes showed higher evolutionary rates (*Dn*/*Ds* = 0.3793). Most protein‐coding genes experienced relaxed purifying selection, and the high *Dn* value (0.1927) indicated that gene functions adjusted to environmental changes effectively. Our findings suggest that gene interval regions play an important role in *Quercus* evolution. We detected greater variation in the intergenic regions (*trnH‐psbA*, *trnK_UUU‐rps16*, *trnfM_CAU‐rps14*, *trnS_GCU‐trnG_GCC*, and *atpF‐atpH*), intron losses (*petB* and *petD*), and pseudogene loss and degradation (*ycf15*). Additionally, the loss of some genes suggested the existence of gene exchanges between plastid and nuclear genomes, which affects the evolutionary rate of the former. However, the connective mechanism between these two genomes is still unclear.

## Graphical abstract

In this paper, our research object is 18 species of *Quercus*, which is a better forest building tree species. We first sequenced the whole cp genome of *Quercus fabri*, *Q. semecarpifolia*, *Q. engleriana*, and *Q. phellos*and then compared it with 14 other *Quercus* cp genomes to explore their evolutionary patterns. We found that most protein‐coding genes have experienced relaxed purifying selection, and discussed the factors related to the evolution rate.

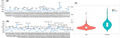

## 1 INTRODUCTION

Nucleotide substitution is a driving force of genome evolution, as the production of nonsynonymous substitutions may change protein functions, which may be fatal. Consequently, natural selection tends to delete these harmful mutations, resulting in most species being under negative selective pressure (Susann et al., 2013; Wang et al., 2018). Evolutionary rates of species may be affected by gene or protein function, selective pressure, population size, generation time, DNA‐repair efficiency, species diversity, and individual species size (Bromham et al., 2015; Hao et al., 2018; Wang et al., 2015). Selective pressure on functional genes has been found to be especially significant in determining rates of protein evolution (Minias et al., 2018; Xing & Lee, 2005). For example, selective pressures associated with habitats appear to have caused the rapid evolution of genes involved in cold response in *Cardamine* (Ometto et al., 2012). Another example are parasitic plant species, which have greater evolutionary rates in their plastid, mitochondrial (mt), and nuclear genomes when compared with other plants as they are deemed to be under greater selective pressure (Bromham et al., 2013). With the rapid development of DNA sequencing technology, it is now possible to study the role of selective pressure in molecular evolution.

Plastids are semiautonomous organelles and include chloroplasts, leucoplasts, chromoplasts, and amyloplasts, which all develop from proplastids of an embryo and have an identical genome. Over the past few decades, plastomes have begun to be used extensively in studying species evolution, migration, identification, and classification (Jose et al., 2015; Moore et al., 2010). Earlier plastome studies mainly focused on comparisons of the absolute rates of evolution of some functional genes, and these results were based on different genes in different species, which may have led to statistical errors (Kenneth et al., 1987). Later, comparisons of evolutionary rates among plastid, mt, and nuclear genomes, as well as between gymnosperms and angiosperms, were conducted (Petersen et al., 2019; Smith et al., 2014). Modern sequencing technology now allows the rapid generation of genomic data, which is essential for a more accurate analysis of factors affecting molecular evolution.

The genus *Quercus* L. is native to the Northern Hemisphere and contains ~500 species and is widely distributed in Asia, northern Africa, Europe, and America (Aldrich & Cavender‐Bares, 2011). Infrageneric classification of *Quercus* is controversial mainly because convergent morphological evolution is commonplace within the genus (Denk et al., 2017) and many morphological differences are a result of introgression (Curtu et al., 2007; Moran et al., 2012). Species of *Quercus* (commonly referred to as oaks) are wind‐pollinated and unable to discriminate among pollen from other species of the same section. Additionally, there is incomplete reproductive isolation among oaks, and as a result, phenotypes of progeny produced by interspecific hybridization are variable and difficult to discern (Williams et al., 2010).

Based on morphological characteristics, Chinese oaks have been classified into two subgenera: *Quercus* subg. *Quercus* and *Q*. subg. *Cyclobalanopsis* (Oerst.) C.K. Schneid. (Zhou, 1992), or these have been considered as distinct genera in the Flora of China (Huang et al., 1999). More recently, phylogenetic research has been conducted on *Quercus* and *Cyclobalanopsis* Oerst. using nuclear DNA (nrDNA) and plastid DNA fragments (Denk et al., 2017) and, most recently, whole plastome, mt, and nuclear genome data (Hipp et al., 2020). In the most recent classification based on the nrDNA and plastid DNA (reviewed in Denk et al., 2017), *Quercus* is divided into two subgenera, *Q*. subg. *Quercus* and *Q*. subg. *Cerris* (Oerst.), corresponding to the New‐ and Old‐World oaks, respectively, and with *Cyclobalanopsis* placed within *Q*. subg. *Cerris* Oerst.. This was corroborated by whole plastome, mt, and nuclear genome data (Hipp et al., 2020). However, relationships within the Old‐World oaks of *Q*. subg. *Cerris* Oerst. are complex. Phylogenetic relationships established by nrDNA tended to differ from those based on plastid data, especially with regards taxa previously circumscribed in *Q*. sect. *Ilex* (reviewed in Denk et al., 2017). Chinese taxonomists continue to accept *Cyclobalanopsis* as a distinct subgenus or genus (Deng et al., 2014; Pu et al., 2002; Zhou, 1992), and controversy also surrounds whether taxa pertaining to *Q*. sect. *Ilex* are a monophyletic lineage (Denk et al., 2017).

In our study, we sequenced the whole plastomes of *Quercus fabri*. Hence, *Quercus semecarpifolia* Smith, *Quercus engleriana* Seem, and *Quercus phellos* L. *Q fabri*, of *Q*. subg. *Quercus* sect. *Quercus*, is a deciduous forest species endemic to China. It is widely distributed south of the Yangtze river and grows on hills or mountains between 50 and 1,900 m a.s.l. (Li, Li, & Fang, 2018; Li, Li, Zang, et al., 2018). *Q. semecarpifolia* and *Q. engleriana*, of *Q*. subg. *Cerris* sect. *Ilex*, are evergreen forest species endemic to China. *Q*. *semecarpifolia* has a narrow distribution, being found only in western China, while *Q. engleriana* has a comparatively large distribution, with its range covering the Chinese provinces Shanxi, Jiangxi, Fujian, Hunan, Hubei, Guangxi, Sichuan, Guizhou, Yunnan, and Tibet (Tang, 2015). *Quercus phellos*, of *Q*. subg. *Quercus* sect. *Lobatae*, is a North American deciduous red oak introduced to China and used extensively in landscape gardening (Chen et al., 2013).

All three Chinese endemic oak (*Q. fabri*, *Q. semecarpifolia*, and *Q. engleriana*) species are ecologically and economically important, and their physiology, genetic diversity, breeding, forest management, and food processing are under intense investigation (Wangda & Oshawa, 2006; Wei et al., 2021). Additionally, there are no native species of *Q*. sect. *Lobatae* in China, and no plastomes sequenced from this section prior to our study, with *Q. phellos* critical to our study for including section *Lobatae* characteristic. The whole plastomes of these four species, coupled with those of 14 other *Quercus* species retrieved from GenBank, belong to the two subgenera and five of the eight sections currently accepted, that is, *Q*. subg. *Quercus* sect. *Quercus*, sect. *Lobatae,* and *Q*. subg. *Cerris* sect. *Cerris*, sect. *Cyclobalanopsis*, sect. *Ilex* (Denk et al., 2017; Hipp et al., 2020). These species’ distributional ranges and habitats largely differ and thus form ideal material for studying evolution of the *Quercus* genus.

The purposes of this study are to (a) generate a well‐resolved phylogenetic framework based on plastomes; (b) analyze the sequence variation of coding and noncoding plastid gene regions and transfer of plastid genes to understand the diversification patterns of *Quercus*; and (c) analyze the selective pressure that acts on essential protein‐coding genes to explore the variation and patterns in evolutionary rates of *Quercus* plastomes.

## 2 MATERIALS AND METHODS

### 2.1 Sampling, DNA extraction, Illumina sequencing, and assembly

Fresh leaves were collected from four *Quercus* species (*Q*. *fabri*, *Q*. *semecarpifolia*, *Q. engleriana*, and *Q. phellos*), kept on ice, and then stored at −80°C until further use. The four oak species were collected from three Chinese provinces (Jiangsu, Yunnan, and Zhejiang; see Table S1 for collection information). Voucher specimens were deposited at the Nanjing Forestry University Herbarium (accession numbers: NJFU‐QF20180501, NJFU‐QS20201001, NJFU‐QE20201002, and NJFU‐QP20201003, respectively).

Genomic DNA was isolated using the modified cetyltrimethylammonium bromide method (Doyle, 1987). A 3 mg of leaf tissue was ground with the addition of 0.35 times the volume of absolute ethanol and inverted several times. Following this, 700 μl of preheated 2% CTAB extract was added to the solution, which was then kept at 65°C in a water bath for 30 min. This solution was then centrifuged at 12,000 r/min for 5 min, after which an equal volume of phenol/chloroform/isoamyl alcohol (25:24:1) was added to the supernatant. This was then centrifuged at 12,000 r/min for 10 min, after which an equal volume of chloroform/isoamyl alcohol (24:1) was added to the supernatant. The supernatant was then transferred to an equal volume of prechilled isopropanol and let stand at −20°C for at least 40 min. The precipitate was then collected, 1 mol/L NaCl 400 μl was added for dissolution, then an equal volume of chloroform/isoamyl alcohol was added and the solution was centrifuged at 12,000 r/min for five minutes. Following this, a 1/2 volume of NaCl solution and 2–2.5 times volume of precooled absolute ethanol was added to the supernatant, followed by gentle shaking until white flocculent precipitate appeared. The precipitate was then collected and washed twice with 70% ethanol, before being placed on the clean bench and blow dried. Agarose gel electrophoresis and spectrophotometry (OD‐1000; Shanghai Cytoeasy Biotech Co., Ltd., Shanghai, China) were used to determine DNA integrity and quality.

The DNA was fragmented with a Covaris sonication device and then the fragments were purified, end‐repaired, and A‐tailed. Adapters were lighted through a 3′‐thymine overhang. Next, the fragment size was selected by agarose gel electrophoresis, and the sequencing library was formed by PCR amplification. Finally, the constructed library was inspected. Sequencing was performed on an Illumina HiSeq 2500 platform (Illumina, Nanjing, China), yielding at least 8.18–10 GB of clean reads.

We selected the plastome sequence of *Quercus aliena* as a reference, and then employed reads to produce two‐way extensions through overlap using NOVOPlasty software (Dierckxsens et al., 2017). When the assembly results were within the expected range, the overlap was greater than 200 bp, and the assembly formed a ring.

### 2.2 Annotation and analysis of the plastid DNA sequences

Genome annotation was performed using CpGAVAS (Chang et al., 2012), with DOGMA (http://dogma.ccbb.utexas.edu/) and BLAST used to confirm the annotation results. tRNAscanSE was used to identify the tRNAs (Schattner et al., 2005). Circular gene maps of *Q. fabri*, *Q. phellos*, *Q. engleriana*, and *Q*. *semecarpifolia* were drawn using the OGDRAWv1.2 program (http://ogdraw.mpimp‐golm.mpg.de/; Lohse et al., 2007). Relative synonymous codon usage was examined using CodonW (Peden, 2000). The annotated plastome sequences of *Q. fabri*, *Q. phellos*, *Q. engleriana,* and *Q*. *semecarpifolia* were deposited in the NCBI Sequence Read Archive (Accession numbers MK693136, MZ196210, MZ196209, MZ196211, respectively).

### 2.3 Genome structure analysis and genome comparisons

The mVISTA program in Shuffle‐LAGAN mode was used to compare the *Q. fabri*, *Q*. *phellos*, *Q. engleriana*, and *Q*. *semecarpifolia* plastomes with the 14 other *Quercus* plastomes (*Q*.*baronii* Skan, *Quercus dolicholepis* A. Camus, *Quercus acutissima* Carruth, *Quercus variabilis* Blume, *Quercus tarokoensis* Hayata, *Quercus edithiae* Skan, *Quercus glauca* Thunb., *Quercus sichourensis* Hu, *Quercus aquifolioides* Rehd. and Wils, *Quercus spinosa* David ex Franchet, *Quercus tungmaiensis* Y.T. Chang, *Q*. *aliena* var. *acutiserrata* Maximowicz ex Wenzig, *Q. aliena* Blume, and *Quercus rubra* L.; Table 1), using the *Q. aliena* annotation as a reference (Mayor et al., 2000). MAUVE v2.4.0 software was used to compare the genes and sequences of the de novo assembled *Q. fabri* plastome sequence, with the *Q. aliena* plastome sequence as the reference (Doose et al., 2017). Geneious Pro v9.1.6 was used to characterize the plastomes of the 18 species and summarize the features into tables (Matthew et al., 2012).

**TABLE 1:** Basic features of the *Quercus* species chloroplast genomes

Species	Accession No.	Length (bp)	GC content (%)	LSC length (bp)	SSC length (bp)	IR length (bp)	Gene number
*Quercus acutissima*	MH607377	161,124	36.8	90,423	19,069	25,816	135
*Quercus aliena*	KU240007	161,150	36.8	90,444	19,054	25,826	126
*Quercus aliena* var*. acutiserrata*	KU240008	161,153	36.8	90,457	19,044	25,826	126
*Quercus aquifolioides*	KX911971	161,225	36.8	90,535	19,000	25,845	126
*Quercus baronii*	KT963087	161,072	36.8	90,341	19,045	25,843	126
*Quercus dolicholepis*	KU240010	161,237	36.8	90,461	19,048	25,864	126
*Quercus edithiae*	KU382355	160,988	36.9	90,351	18,954	25,842	128
*Quercus fabri*	MK693136	161,250	36.8	90,563	18,995	25,846	136
*Quercus glauca*	NC_036930	160,798	36.9	90,229	18,906	25,831	134
*Quercus rubra*	JX970937	161,304	36.8	90,542	19,025	25,869	129
*Quercus sichourensis*	NC_036941	160,681	36.9	90,154	18,857	25,835	134
*Quercus spinosa*	KX911972	161,156	36.8	90,441	18,997	25,859	126
*Quercus tarokoensis*	NC_036370	161,355	36.8	90,602	19,033	25,860	134
*Quercus tungmaiensis*	NC_036936	160,702	36.9	90,113	18,939	25,825	134
*Quercus variabilis*	KU240009	161,077	36.8	90,387	19,056	25,817	126
*Quercus phellos*	MZ196210	161,331	36.8	90,550	19,061	25,860	127
*Quercus engleriana*	MZ196209	161,053	36.8	90,356	18,943	25,877	133
*Quercus semecarpifolia*	MZ196211	161,312	36.8	90,505	19,077	25,865	133

### 2.4 Phylogenetic analyses

We used 67 shared protein‐coding sequences of the 18 *Quercus* species and two outgroup species, *Malus prunifolia* (Willd.) Borkh. (NC_031163) and *Ulmus gaussenii* W. C. Cheng (NC_037840), to build the phylogenetic trees. Phylogenetic relationships were constructed using the BI analysis in Mrbayes v 3.2.6 (Huelsenbeck & Ronquist, 2001). First, the sequences were aligned in MAFFT (Kazutaka & Standley, 2013). Then, the multiple sequence alignment was visualized and manually adjusted using BioEdit (Hall, 1999). We used JmodelTest2 to evaluate the best‐fitting models of nucleotide substitution (Darriba et al., 2012). GTR +G was selected as the best substitution model for BI analyses.

### 2.5 Gene selective pressure analysis

The 79 shared protein‐coding genes of the 18 *Quercus* species, which had sequence lengths longer than 300 bp were extracted and aligned separately using Mega 7.0 to analyze synonymous substitution rates (*Ds*) and nonsynonymous substitution rates (*Dn*) using the codeml program in PAML package version 4.7.1 under a one‐ratio branch model (Yang & Yang, 2007). We constructed the tree topology using BI as the tree file. After removing outliers, a total of 64 effective genes were included in statistical analyses using SPSS 19.0 software (SPSS, Inc., Chicago, IL, USA). The *Ds*, *Dn*, and *Dn*/*Ds* ratio of functional gene functional groups were compared by ANOVA. A Mann–Whitney U test of two independent samples was used to determine whether there were significant differences in *Ds*, *Dn*, and *Dn*/*Ds* among the *Quercus* species and previously published values of gymnosperms (Wang et al., 2015) based on the calculated *P*‐values obtained from the double‐tail test (*p* < .05).

## 3 RESULTS

### 3.1 Plastome assembly and characteristics

**FIGURE 1:**
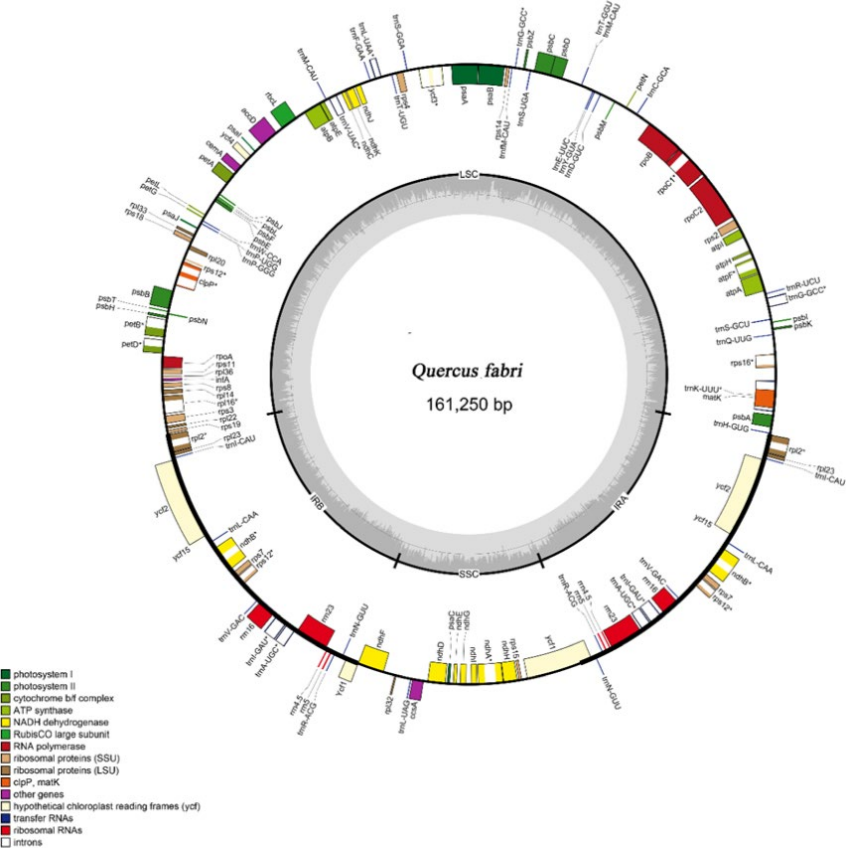
Gene map of the *Quercus fabri* plastome. Genes displayed outside the circle are transcribed clockwise, while internal transgenes are transcribed counterclockwise. Genes belonging to different functional groups are color‐coded. The dashed line region in the inner circle represents the GC content of the plastome

The *Q. phellos* complete plastome size was the largest with 161,331 bp (Table 1). The four oak species displayed a typical quadripartite structure, and we chose to describe the *Q. fabri* plastome, which included a pair of inverted repeats (IRs; 25,846 bp) separated by a single large copy (LSC; 90,563 bp) and a single small copy (SSC; 18,995 bp) region (Figure 1; Table 1). The *Q. fabri* genome contained the most functional genes (about 136), including 89 protein‐coding genes, 39 tRNA genes, and eight rRNA genes (Tables 1 and S1). Introns occurred in 12 genes, *rps16*, *atpF*, *rpoC1*, *ycf3*, *clpP*, *petB*, *petD*, *rpl16*, *rpl2*, *ndhB*, *ndhA,* and *rpl2* (Table S2). Of these, the *rps12* gene was unique as it contained three exons, one of which occurred in the LSC region, while the other two exons occurred in the IRa and IRb regions. We found 26,756 codons in all the coding sequences (Table S3). Leucine was the most common amino acid, encoded by 10.84% (2,903) of the codons, while cysteine was the least frequent amino acid, encoded by 1.35% (331). The A‐ and U‐ending codons occurred at high frequencies, and all types of preferred synonymous codons (relative synonymous codon usage >1) ended with A or U.

There was no gene rearrangement among the 18 species during the evolution of *Quercus* (Figure S1). We compared the details of four junctions (LSC/IRA, LSC/IRB, SSC/IRA, and SSC/IRB) among the plastomes of the 18 species (Figure S2). At the SSC/IRA boundary, the *ycf1* gene ranged from 4,600 (*Q. rubra*) to 4,635 bp (*Q. tungmaiensis*) in the SSC region. Among the 18 oak species, the length of the *ndhF* and *ycf1* genes of *Q. engleriana* showed obvious differences from the others. A range of 72–82 simple sequence repeats (SSRs) were detected in the plastomes of the 18 *Quercus* species (Figure S3).

### 3.2 Phylogenetic analysis

**FIGURE 2:**
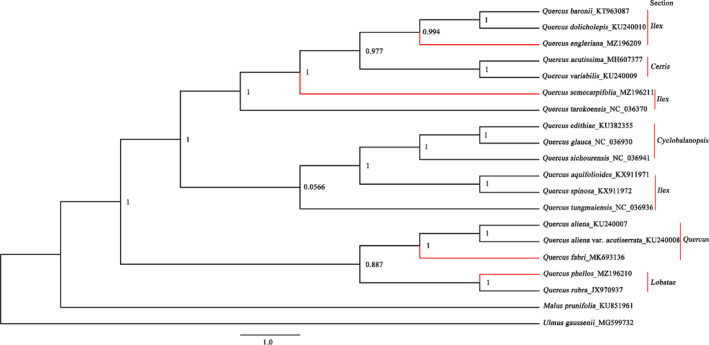
Maximum‐likelihood phylogenetic tree constructed using 67 shared protein‐coding sequences of plastomes of 18 *Quercus* species. Numbers above the lines indicate the likelihood bootstrap values. *Malus prunifolia* and *Ulmus gaussenii* were used as the outgroups

The phylogenetic relationships of the 18 *Quercus* species, constructed using 67 shared protein‐coding sequences, were strongly supported and can be seen in Figure 2. *Quercus* was found to be divided into two major evolutionary clades. The first clade included taxa of *Quercus* subg. *Cerris* sections *Ilex*, *Cerris*, and *Cyclobalanposis*, while the second included taxa of *Quercus* subg. *Quercus* sections *Quercus* and *Lobatae*, as defined by Denk et al., 2017). *Quercus* subg. *Cerris* sect. *Ilex* was not retrieved as a monophyletic group due to the insertion of taxa from sect. *Cerris* and sect. *Cyclobalanposis*, which divided sect. *Ilex* into three strongly supported polyphyletic evolutionary clades. Taxa of *Q*. subg. *Quercus* sect. *Quercus* (*Q. aliena*, *Q. aliena* var. *acuteserrata, Q. fabri*) formed a well‐supported monophyletic clade that was sister to a clade comprising taxa of *Q*. subg. *Quercus* sect. *Lobatae* (*Q*. *rubra* and *Q. phellos*).

### 3.3 Deletion and transfer of plastid genes

The variation in plastome length among the 18 oak species studied was small, with plastomes ranging from 160,681bp (*Q. sichourensis*) to 161,355 bp (*Q. tarokoensis*; Table 1). Genes were consistently distributed among the different genomic regions across the 18 species. rRNA genes showed the most remarkable conservation, with all species containing eight rRNA genes. The number of protein‐coding genes differed among the *Quercus* species; however, they shared 79 common genes. In this study, *Q. rubra*, *Q. edithia,* and *Q. sichourensis* lost the *psbB, rpl22,* and *ycf4* genes, respectively. Additionally, only seven species (*Q. edithiae*, *Q. fabri*, *Q. rubra,* and *Q. acutissima*, *Q. phellos*, *Q. engleriana*, and *Q. semecarpifolia*) contained the *ycf15* gene.

**FIGURE 3:**
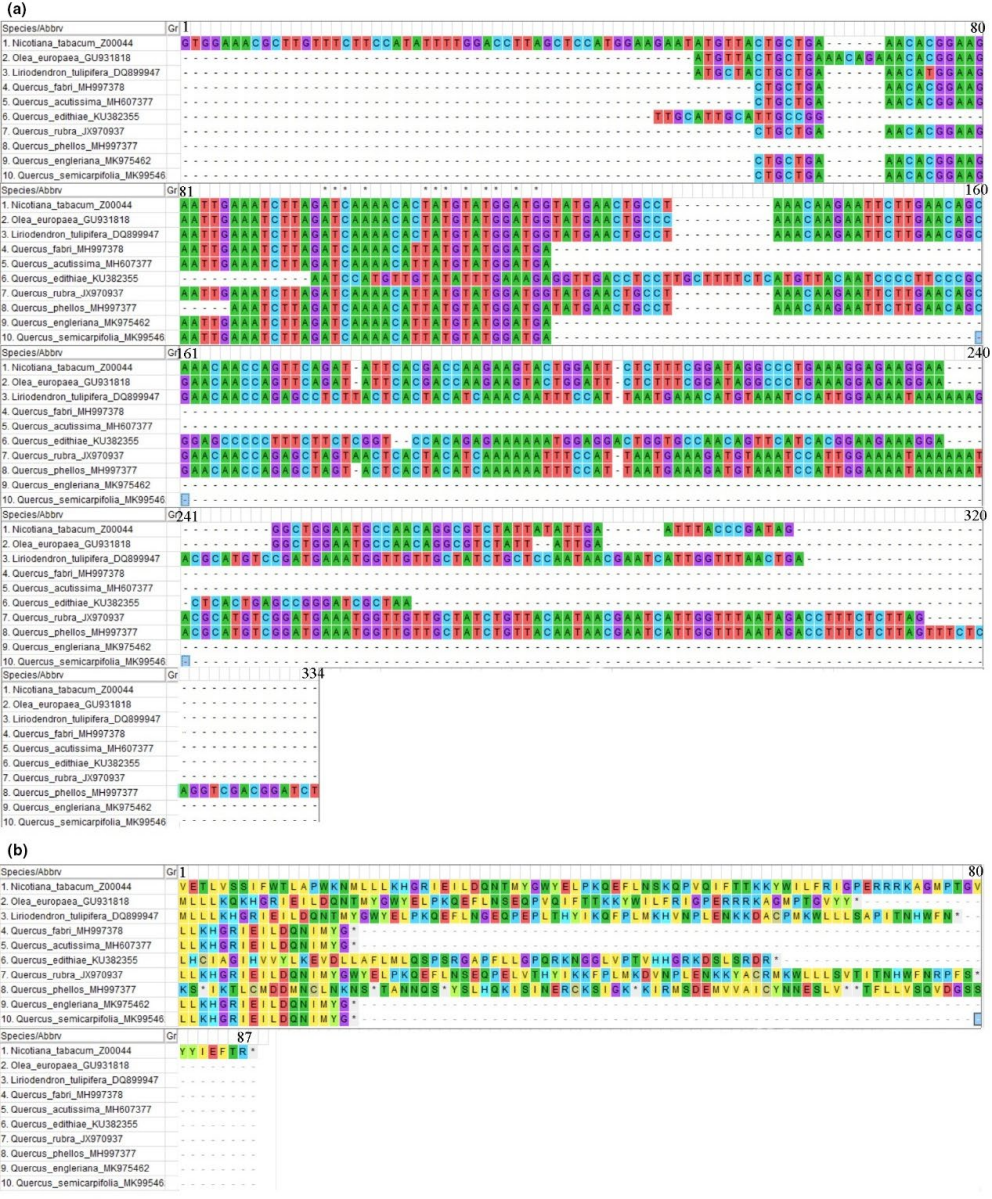
Alignment of the *ycf15* gene and protein sequences from the 18 *Quercus* species. a. Alignment of the *ycf15* gene sequences; b. Alignment of the *ycf15* protein sequences

We used the *ycf15* gene sequence of *Nicotiana tabacum* Fischer ex Lehmann. as a reference, as well as those of *Olea europaea* Linn. and *Liriodendron tulipifera* Linn., in comparisons with the seven *Quercus* species (*Q. acutissima*, *Q. fabri*, *Q. edithiae*, *Q. rubra*, *Q. phellos*, *Q. engleriana*, and *Q. semecarpifolia*) containing *ycf15* (Figure 3). In *N. tabacum* (Z00044) and *O. europaea* (GU931818), the *ycf15* gene is complete, while in *L. tulipifera* (DQ899947), the *ycf15* gene is a pseudogene. Unlike the *ycf15* sequence in most species, these seven species employed CTG and TTG start codons instead of ATG and GTG. Compared with the representative species, the *ycf15* genes in *Q. rubra*, *Q. phellos*, and *Q. edithiae* were similar to that of *L. tulipifera*, indicating that they are pseudogenes. The lengths of the *ycf15* gene in the remaining four species (*Q. fabri*, *Q. acutissima*, *Q. engleriana*, and *Q. semecarpifolia*) were only ~50 bp, indicating pseudogene degeneration. These seven species belong to different subgenera and sections, and their *ycf15* gene sequences are very different.

### 3.4 Comparative analysis of coding and noncoding regions

**FIGURE 4:**
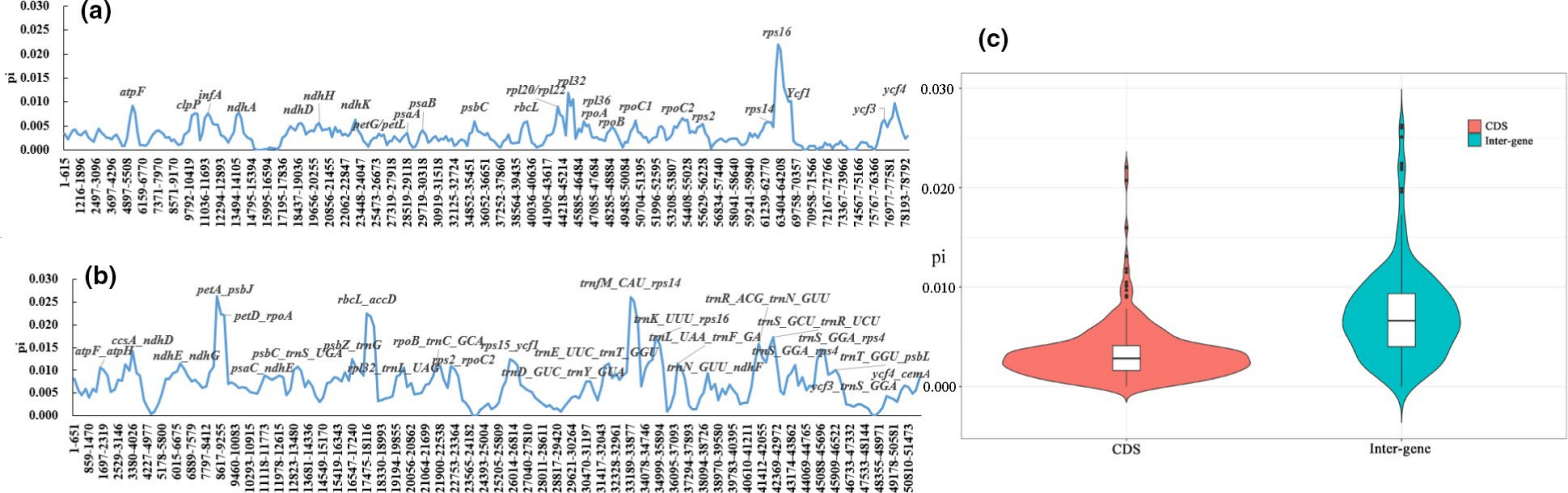
Percentages of variable characters in 18 aligned *Quercus* plastomes. (a) Coding regions; (b) Noncoding regions; (c) Analysis of the differences between coding and intergenic region. *X‐axis*: position in a window. *Y‐axis*: nucleotide diversity of each window

Using the mVISTA program (Figure S4), we found that conserved noncoding sequences (containing intron sequences) had greater variation levels than exon sequences. The sequences of noncoding regions which had large variations included *trnH_GUG‐psbA*, *trnK_UUU‐rps16*, *rps16‐trnQ_UUG*, *trnS_GCU‐trnG_GCC*, *atpF‐atpH*, *atpI‐rps2*, *psbM‐trnD_GUC*, *trnM_CAU‐psbD*, *psbZ*‐*trnG_UCC*, *trnfM_CAU‐rps14*, *trnF_GAA‐ndhJ*, *ndhC*‐*trnV_UAC*, *rbcL‐accD*, *ycf4*‐*cemA*, *ccsA‐ndhD*, and *rpl32‐trnL_UAG*. We analyzed the coding and noncoding regions’ sequences using a sliding window to present the observed variations using digitization. The sliding window analysis showed that protein‐coding genes which had large pi values were: *atpF*, *clpP*, *infA*, *ndhA*, *ndhD*, *ndhK*, *ndhH*, *psbC*, *rpl20*, *rpoC2*, and *rps16* (Figure 4a). Among these genes, those with introns showed the greatest variation. The following genes had sequence variation in their gene interval regions: *trnK_UUU‐rps16*, *petA‐psaJ*, *rbcL‐accD*, *trnfM_CAU‐rps14*, and *trnS_GUC*‐*trnR_UCU* (Figure 4b). The sequence variation in protein‐coding genes and gene interval regions reflected by the sliding window was the same as those determined using the mVISTA software. In general, we confirmed that the variations in noncoding sequences are greater than those of coding sequences through different analyses (Figure 4c).

**FIGURE 5:**
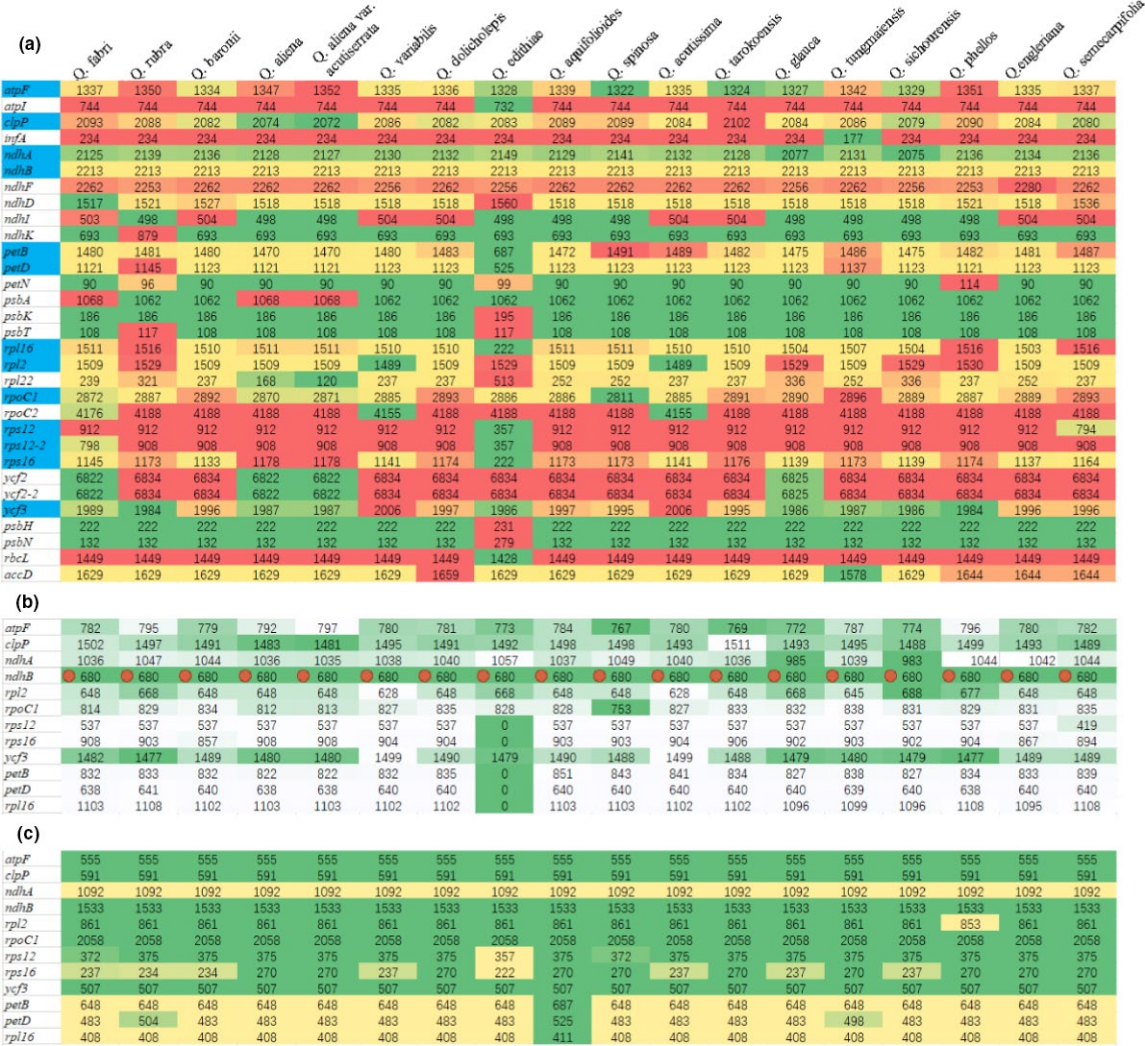
Analysis of variations in protein‐coding gene lengths. (a) A total of 31 protein‐coding genes with variable gene lengths; (b) Length variations of introns (noncoding sequences) in 12 protein‐coding genes; (c) Length variations of exons (coding sequences) in 12 protein‐coding genes. Different colors in the same row represent changes in length

To further explore the sequence variation characteristics of coding and noncoding regions, we counted the shared coding‐protein genes of the 18 *Quercus* species studied. These species shared 79 protein‐coding genes, 6 of which (*ndhB*, *rpl2*, *rps12*, *rps7*, *ycf2,* and *rpl23*) had duplicate copies. Of the 79 protein‐coding genes, 31 had variable lengths (Figure 5a). We analyzed the lengths of these 31 protein‐coding genes and found 12 had introns, with larger length variations than exons, such as *atpF*, *clpP*, *ndhA,* and *rpl2*. The intron lengths of the 31 protein‐coding genes also differed. Additionally, *Q. edithiae* lost intron regions in *petB*, *petD*, *rps12*, *rps16*, and *rpl16*, which caused variation in intron lengths (Figure 5b). The sequence lengths of exons of the 31 protein‐coding genes also varied, such as those of *rps16*, *rps12*, *petD*, *petB,* and *rpl16* (Figure 5c). Protein‐coding genes without introns were relatively conserved, with limited variation in gene lengths. Still, there were also several unique genes, such as *rpl22*, *rpoc2,* and *ycf2*, which had lengths that fluctuated greatly among the 18 species.

### 3.5 Gene selective pressure analysis

**FIGURE 6:**
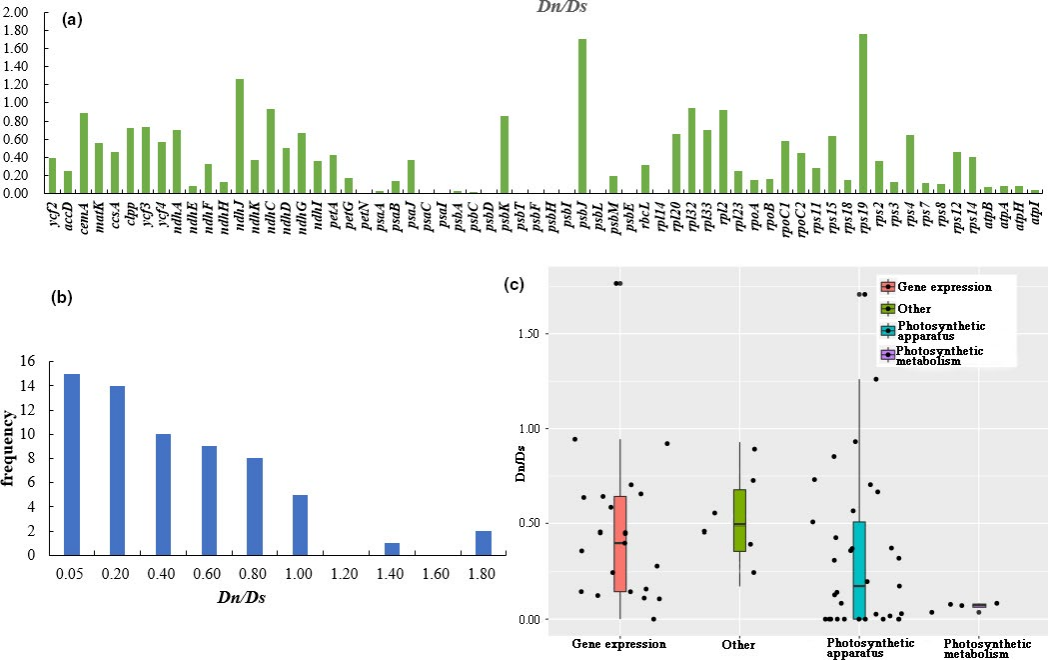
Evaluation of differences in plastid gene substitution rates in *Quercus*. (a) *Dn*/*Ds* distribution map of protein‐coding genes; (b) *Dn*/*Ds* frequency distribution of protein‐coding genes; (c) Analysis of *Dn*/*Ds* variations in different functional genes

We divided the 64 protein‐coding genes into four major categories: Photosynthetic metabolism; Photosynthetic apparatus; Gene expression; Other (Table 2). After removing the “other” category, the data presented in Table 2 revealed that “Gene expression” had the highest average synonymous and nonsynonymous substitution rates (*Ds* *= *0.3108 and *Dn* *= *0.2095, respectively), while “Photosynthetic metabolism” had the lowest *Ds* and *Dn* values of 0.0412 and 0.0027, respectively. When comparing *Ds*, *Dn,* and evolutionary rates (*Dn*/*Ds*) of the four gene categories, ANOVA found no significant differences (*p* > .05, Table 2), which may be due to the small sample size, leading to a lack of statistical validity. To estimate selective pressure, we studied the average evolutionary rates (*Dn*/*Ds*) of different genes (Figure 6). The value of *Dn* or *Ds* of some genes was equal to 0.0, so *Dn*/*Ds* value could not be calculated. The *Dn*/*Ds* ratio of the 18 species ranged from 0.0361 to 1.7654, varying by up to a factor of 49 (Figure 6a). With the exception of three genes (*ndhJ*, *psbJ*, and *rps19*), the *Dn*/*Ds* ratios of the remaining genes were all less than 1, suggesting that most protein‐coding genes in *Quercus* are under purifying selection (Figure 6b). Of these, we found the variation of evolutionary rate within gene groups is still considerable, with some genes with unknown functions evolving faster than other genes (Figure 6c). Compared with gymnosperms, *Quercus* has higher *Dn* (*p* < .05) and *Dn*/*Ds* (*p* > .05) ratios (Table S4).

**TABLE 2:** Substitution and evaluation rates of *Quercus* plastid genes (standard deviation) using *p* < .05 as significant criterion

Categories	*Dn*	*Ds*	*Dn*/*Ds*
Photosynthetic metabolism	0.0027 (0.0023)	0.0412 (0.0281)	0.0674 (0.0214)
Photosynthetic apparatus	0.1496 (0.5687)	0.2403 (0.7891)	0.3302 (0.4131)
Gene expression	0.2095 (0.6143)	0.3108 (0.9067)	0.4684 (0.4048)
Other	0.4978 (1.1837)	0.6953 (1.6252)	0.5454 (0.2334)
Average	0.1927 (0.6384)	0.2936 (0.8988)	0.3793 (0.3747)
*F*	0.619	0.534	1.777
*p*	.606	.661	.161

## 4 DISCUSSION

The genus *Quercus* has become an established member of the plant communities of the Northern Hemisphere. It is considered to have arisen during the Paleogene (Barrón et al., 2017; 56 Ma according to Hipp et al., 2020) and subsequently underwent deep geographic separation of major clades within the first 15 Myrs after origin of the genus (Hipp et al., 2020). It is then hypothesized to have spread to a diverse range of environments in the later Cenozoic (Barrón et al., 2017), with China now forming the second‐largest diversity center of *Quercus* (Oldfield & Eastwood, 2007). Their high diversity and remarkable introgression make *Quercus* a good study‐system for understanding the link between the prevalence of adaptive gene flow and the evolutionary history of forest trees. Over the past five years, much research has focused on reexamining the oak phylogeny and investigating the history of diversification across the genus (Deng et al., 2018; Denk et al., 2017; Hipp et al., 2020; Jiang et al., 2019). The topology of our phylogenomic tree matches previous analyses based on plastid and nrDNA gene fragments and RAD‐seq of whole plastid, mitochondrial, and nuclear genomes (reviewed in Denk et al., 2017; Hipp et al., 2020) for all sections except that of sect. *Ilex*. In previous analyses (Denk et al., 2017; Hipp et al., 2020), sect. *Ilex* was retrieved as a monophyletic group, sister to sect. *Cerris*. However, in our study, sect. *Ilex* was polyphyletic and divided into three strongly supported evolutionary clades placed in‐between sect. *Cyclobalanopsis* and sect. *Cerris*. This may be related to differential rates of introgression, which make it difficult for us to establish completely reliable phylogenomic relationships based on only one genome (plastome) and a relatively small sample size. The focus of future phylogenomic studies of *Quercus* should be on incorporating a more comprehensive sampling of whole genomes, as well as including other molecular sampling approaches such as nuclear genome skimming, that covers all sections and morphological and biogeographic variability present in the genus. This should include whole plastomes and nrDNA from taxa belonging to *Q*. subg. *Quercus* sect. *Protobalanus*, *Ponticae*, *Virentes*, which were lacking in this study.

Compared with gymnosperm genera, *Quercus* has a much higher species diversity, and thus it is assumed a higher genetic diversity, albeit with extant species having a similar recent evolutionary age (Nagalingum et al., 2011). Thus, the question arises whether differences in the evolutionary patterns of plastomes affect their genetic diversity? Based on our results, we hypothesize that, during its early evolutionary history, *Quercus* had a considerable *Dn* value that enabled gene functions to be effectively adjusted to environmental changes occurring over the last c. 56 Ma (Hofmann et al., 2011), with a substantial body of research also supporting this view. For example, there are significant differences in the phenology, flowering biology, and other characteristics of oak species in accordance with their environment. The flowering time of oak species in temperate regions is in the spring, while tropical areas are in the dry season (Ducousso et al., 1993). Northern temperate deciduous forest species also have a higher pollen volume than that of earlier diverging evergreen oak species from warmer and drier climates, with the high pollen volume likely an adaptation evolved to cope with the relatively adverse damper cooler climate which inhibits anemophily (Cao & Zhou, 2002; Nagalingum et al., 2011). Additionally, most protein‐coding genes large *Dn*/*Ds* values further indicate that most genes have experienced relaxed purifying selection and that natural selection allowed *Dn* mutations of these genes. The *Dn*/*Ds* values of the three genes, *ndhJ*, *psbJ*, and *rps19*, were greater than 1, indicating positive selection, which is evidence of the proteins’ adaptive evolution. The evolutionary rates of genes which have varied functions are also different. However, the question arises as to why do genes involved in photosynthetic metabolism evolve more slowly than other genes? We speculate that selective constraints may cause differences in gene function and expression. *atpA*, *atpB*, and *atpE* encode the α, β, and ε subunits of plastid ATPase, respectively. ATPase is the critical enzyme in energy metabolism and plays a central role in photosynthesis (Felix et al., 2020). The divergence of *Dn* is proof that genes go through different degrees of selective constraints, but it is still challenging to discern what degree of selective constraints caused this difference.

Numerous studies of angiosperm plastomes have found that most variation occurred in noncoding sequences, and occasionally in the protein‐coding sequences which evolve more rapidly. Additionally, a large portion of conserved noncoding DNA appears to be under similar selective constraints as protein‐coding DNA but, until now, most studies on molecular evolution have focused on protein‐coding sequences (Chen & Blanchette, 2007; Xie et al., 2018). In this study, the degree of variation in the noncoding sequences (gene spacing regions) was greater than that in the protein‐coding sequences. Insertion and deletion mutations lead to microstructural changes, which can be coded as evolutionary events during phylogenomic analyses. Even in the coding region, introns do not encode proteins, and 20%–68% of introns are species‐specific. Over the past few hundred million years, introns have been gained and lost, although during the evolution of plants, introns have mainly been lost (Roy & Penny, 2007), while in algae abundant introns have been lost and gained recently (Turmel et al., 2017). In our study, we found that *Quercus* has 12 introns, with only *Q. edithiae* having lost intron regions in *petB*, *petD*, *rps12*, *rps16*, and *rpl16*. This could have occurred as a result from mistakes during gene annotation. However, this scenario could also represent patterns of evolution in *Quercus*. Differing mutation rate is the main cause of differentiations in evolutionary rate, and most mutation events generally occur in introns, untranslated regions (UTRS), and noncoding regulatory regions. Thus, the number of introns in a genome may reflect the evolutionary rate to a large extent. Species with slower evolutionary rates retain more introns than those with faster evolutionary rates.

Pseudogenes, that is, sequences lacking coding abilities, may also play essential roles in genome evolution (Wang et al., 2012). Redundant DNA deletion events frequently occur in genomes, significantly reducing the pseudogene contents (Lafontaine & Dujon, 2010). Whether the pseudogene *ycf* encodes a protein has been controversial (Shi et al., 2013) and only recently was it confirmed that the basal groups of asterids have complete *ycf15* gene structures and are assumed to have protein‐coding functions (Curci et al., 2015). However, a small number of eudicots have *ycf15* gene structures identified as pseudogenes. These were mainly found in the Ericales, Gentianales, Lamiales, Solanales, and Apiales, which had faster rates of genome evolutionary (Ku et al., 2013). The *ycf15* genes of *Q. edithiae*, *Q. phellos*, and *Q. rubra* have structures similar to those of the species mentioned above (*L. tulipifera*, *Gentianopsis paludosa,* and *Jasminum nudiflorum*). We therefore speculate that their pseudogenes are not encoding genes. In addition to the above‐mentioned *ycf15* pseudogene exhibiting a complete structure, there are few species, such as those belonging to Poaceae, Ranunculaceae, Caryophyllaceae, Cannabaceae, Moraceae, Asteraceae, Lythraceae, and Melastomataceae, in which the *ycf15* gene has undergone genetic degeneration, resulting in its reduction to only c. 50 bp in length (Choi & Park, 2015; Liu et al., 2013; Nie et al., 2012). Pseudogenes are considered to be less influenced by selective pressure during evolution as they can effectively reflect the molecular records of ancestors, which is very helpful in the study of molecular evolution. The question arises as why the fates of the *ycf15* genes in the seven species (*Q. acutissima*, *Q. fabri*, *Q. edithiae*, *Q. phellos*, *Q. engleriana*, *Q. semecarpifolia*, and *Q. rubra*) are so different? This problem may be resolved through the convenience of transcriptome sequencing, which permits mapping the transcriptome reads to the *Quercus* plastome to identify the pattern of gene expression and especially that of the *ycf15* genes. This problem will be gradually solved through whole‐genome sequencing, which will help in understanding the evolution of the *Quercus* plastome.

Interactions among the three genomes (nuclear, plastid, and mitochondrial) in plant cells may also affect the rate of evolution of *Quercus* plastomes. Gene transfers among these three genomes may lead to specific correlations between their evolutionary rates (Goremykin et al., 2009; Jansen et al., 2011; Millen et al., 2001). In angiosperms, *rpl22*, *rpl23*, *rpl32*, *rpl33*, *rps16*, *accD*, *psaI*, *ycf4*, *ycf1*, *ycf2,* and *infA* genes disappeared in some groups, with the disappearance frequency of *infA* gene being highest, although the *infA* gene appeared recently in *Quercus* (Millen et al., 2001). In this study, we identified some gene losses, such as *psbB*, *rpl22*, *ycf4,* and *ycf15*. The *rpl22* gene occurs in the nuclear genome of Fagaceae plants and appeared approximately 34–37 Ma, which explains the gene exchange between plastid and nuclear genes (Jansen et al., 2011). Additionally, DNA replication and repair mechanisms in mitochondrial and plastid genomes have many similarities (Smith et al., 2014). In conclusion, we believe that these three genomes have related evolutionary rates, but the mechanism behind this is still unclear.

## 5 CONCLUSIONS

The whole plastome phylogenomic relationships retrieved in our study mostly coincide with previous research (Denk et al., 2017; Hipp et al., 2020) in their support for sect. *Quercus* and sect. *Lobatae* of subg. *Quercus*, and sect. *Cerris* and sect. *Cyclobalanopsis* of subg. *Cerris*. However, our whole plastome topology does not support findings from Denk et al. (2017) and Hipp et al. (2020) that sect. *Ilex* is a monophyletic group. The polyphyly of sect. *Ilex* is seen by how sect. *Cerris* and sect. *Cyclobalanposis* are inserted between three strongly supported lineages of sect. *Ilex* taxa. Before taxonomic recircumscription of these lineages can be done, phylogenomic studies incorporating a more comprehensive and varied molecular sampling are needed. This is especially pertinent as different genomes, and regions of the genome, can give largely varied interpretations of the evolutionary history of oaks (Hipp et al., 2020).

Based on the whole plastome sequences of 18 *Quercus* species, the evolutionary model of nucleotide substitution rates of typical plastomes found that most protein‐coding genes have experienced relaxed purifying selection. Furthermore, the high *Dn* value indicated that these genes' functions have effectively adjusted to changes in the environment. Noncoding sequences were also found to have more significant variation, including variation in the interpretation of the intergenic region, loss of introns, and loss and degradation of pseudogenes, which are all manifestations of plastid evolution. Additionally, transfers of individual genes between the plastid and nuclear genomes were identified. This allows us to speculate that the evolution of the oak plastome was correlated with that of the other two genomes (nuclear and mitochondrial) to some extent. Continued work focused on a comprehensive molecular sampling of all *Quercus* species, coupled with integrating new whole‐genome sequencing technologies including those focused on the nuclear genome, will be fundamental in improving our understanding of the underlying mechanisms of evolution of both *Quercus* and organisms in general.

## CONFLICT OF INTEREST

None declared.

## AUTHOR CONTRIBUTIONS

**Xuan Li:** Data curation (equal); Formal analysis (equal); Methodology (equal); Resources (equal); Software (equal); Writing‐original draft (equal); Writing‐review & editing (equal). **Yongfu Li:** Formal analysis (equal); Software (equal). **Steven Paul Sylvester:** Writing‐review & editing (equal). **Mingyue Zang:** Formal analysis (equal). **Yousry A. El‐Kassaby:** Supervision (equal); Writing‐review & editing (equal). **Yanming Fang:** Funding acquisition (lead); Resources (lead); Supervision (equal).

## Supporting information

Appendix S1

## Data Availability

All the data are available at Genbank. Genbank accessions MK693136, MZ196210, MZ196209, MZ196211.
