# Supplementary material for: Evolutionary patterns of nucleotide substitution rates in plastid genomes of Quercus
Source: Ecol Evol. 2021 Aug 30;11(19):13401–14. doi: 10.1002/ece3.8063 (PMC8495791; doi:10.1002/ece3.8063)
Supplement: Supplementary file 1 — Appendix S1 [file ECE3-11-13401-s001.docx]

Appendices

Table S1. Information of the four newly sequenced oak species

| Code | Species | Section | Plastome | Location | voucher specimen |
| --- | --- | --- | --- | --- | --- |
| 1 | *Q. fabri* Hence | *Quercus* | MK693136 | Zijin Mountain, Nanjing  (N 32°04′, E 118°50′) | NJFU-QF20180501 |
| 2 | *Q. semecarpifolia* Smith | *Ilex* | MZ196211 | Kunming Botanical Garden, Kunming  (N 25°08′, E 102°44′) | NJFU-QS20201001 |
| 3 | *Q. engleriana* Seem | *Ilex* | MZ196209 | Hangzhou Botanical Garden, Hangzhou  (N 30°15′, E 120°07′) | NJFU-QE20201002 |
| 4 | *Q. phellos* L. | *Lobatae* | MZ196210 | Hangzhou Botanical Garden, Hangzhou  (N 30°15′, E 120°07′) | NJFU-QP20201003 |

**Table S2.** List of genes annotated in the plastome of *Querucs fabri* that were sequenced in this study.

| **Function** | **Genes** |
| --- | --- |
| RNAs, transfer | *trnH-GUG, trnK-UUU*, trnQ-UUG, trnS-GCU, trnG-GCC*, trnR-UCU, trnC-GCA, trnD-GUC, trnY-GUA, trnE-UUC, trnT-GGU, trnM-CAU, trnS-UGA, trnfM-CAU, trnS-GGA, trnT-UGU, trnL-UAA*, trnF-GAA, trnV-UAC*, trnW-CCA, trnP-UGG, trnP-GGG, trnL-CAA, trnN-GUU, trnL-UAG, trnR-ACG, trnA-UGC*, trnI-GAU*, trnV-GAC, trnI-CAU* |
| RNAs, ribosomal | *rrn16* ^a^*, rrn23* ^a^*, rrn4.5* ^a^*, rrn5* ^a^ |
| Transcription and splicing | *rpoC2, rpoC1*, rpoB, rpoA* |
| Translation, ribosomal proteins |  |
| Small subunit | *rps16*, rps2, rps14, rps4, rps18, rps11, rps8, rps3, rps19, rps7, rps12**, rps15* |
| Large subunit | *rpl33, rpl20, rpl36, rpl14, rpl16*, rpl22, rpl2*, rpl23, rpl32* |
| Photosynthesis |  |
| ATP synthase | *atpA, atpF*, atpH, atpI, atpE, atpB* |
| Photosystem I | *psaB, psaA, psaI, psaJ, psaC* |
| Photosystem II | *psbA, psbK, psbI, psbM, psbD, psbC, psbZ, psbJ, psbL, psbF, psbE, psbB, psbT, psbN, psbH* |
| Calvin cycle | *rbcL* |
| Cytochrome complex | *petN, petA, petL, petG, petB*, petD** |
| NADH dehydrogenase | *ndhJ, ndhK, ndhC, ndhB*, ndhF, ndhD, ndhE, ndhG, ndhI, ndhA*, ndhH* |
| Others | *matK, ycf3**, accD, ycf4, cemA, clpP**, infA, ycf2, ycf15, ycf1, ccsA* |

*Genes containing one intron; ** genes containing two introns; ^a^ duplicated genes.

**Table S3.** Codon–anticodon recognition patterns and codon usage in the *Quercus fabri* plastome.

| **Amino Acid** | **Codon** | **No.** | **RSCU** | **tRNA** | **Amino Acid** | **Codon** | **No.** | **RSCU** | | **Trna** |
| --- | --- | --- | --- | --- | --- | --- | --- | --- | --- | --- |
| Ala | GCG | 160 | 0.51 |  | Pro | CCA | 318 | 1.15 | trnP-UGG | |
|  |  |  |  |  |  |  |  |  |  | |
| Ala | GCC | 202 | 0.64 |  | Pro | CCC | 231 | 0.84 | trnP-GGG | |
| Ala | GCU | 552 | 1.74 |  | Pro | CCU | 379 | 1.37 |  | |
| Ala | GCA | 352 | 1.11 | trnA-UGC | Pro | CCG | 177 | 0.64 |  | |
| Cys | UGU | 257 | 1.42 |  | Gln | CAG | 255 | 0.52 |  | |
| Cys | UGC | 104 | 0.58 | trnC-GCA | Gln | CAA | 720 | 1.48 |  | |
| Asp | GAC | 205 | 0.41 | trnD-GTC | Arg | AGA | 501 | 1.85 | trnR-UCU | |
| Asp | GAU | 785 | 1.59 |  | Arg | AGG | 205 | 0.76 |  | |
| Glu | GAA | 1006 | 1.50 | trnE-UUC | Arg | CGU | 302 | 1.12 | trnR-ACG | |
| Glu | GAG | 334 | 0.50 |  | Arg | CGC | 107 | 0.40 |  | |
| Phe | UUU | 969 | 1.24 |  | Arg | CGA | 354 | 1.31 |  | |
| Phe | UUC | 588 | 0.76 | trnF-GAA | Arg | CGG | 154 | 0.57 |  | |
| Gly | GGU | 545 | 1.28 |  | Ser | AGU | 357 | 1.11 | trnS-GCU | |
| Gly | GGG | 325 | 0.76 |  | Ser | AGC | 135 | 0.41 |  | |
| Gly | GGA | 639 | 1.50 |  | Ser | UCU | 523 | 1.61 |  | |
| Gly | GGC | 200 | 0.47 | trnG-GCC | Ser | UCC | 317 | 0.97 | trnS-GGA | |
| His | CAU | 458 | 1.46 |  | Ser | UCA | 402 | 1.24 |  | |
| His | CAC | 170 | 0.54 |  | Ser | UCG | 218 | 0.67 | trnS-UGA | |
| Ile | AUC | 527 | 0.68 | trnI-GAU | Thr | ACU | 464 | 1.51 |  | |
| Ile | AUA | 724 | 0.93 |  | Thr | ACG | 147 | 0.48 |  | |
| Ile | AUU | 1075 | 1.39 |  | Thr | ACC | 236 | 0.77 | trnF-GGU | |
| Lys | AAG | 402 | 0.55 |  | Thr | ACA | 385 | 1.25 | trnT-UGU | |
| Lys | AAA | 1063 | 1.45 |  | Val | GUU | 508 | 1.41 |  | |
| Leu | UUG | 614 | 1.27 | trnL-UAA | Val | GUC | 177 | 0.50 | trnV-GAC | |
| Leu | UUA | 826 | 1.71 | trnL-CAA | Val | GUA | 533 | 1.50 | trnV-UAC | |
| Leu | CUU | 579 | 1.20 |  | Val | GUG | 223 | 0.63 |  | |
| Leu | CUA | 425 | 0.88 | trnL-UAG | Trp | UGG | 475 | 1.0 | trnW-CCA | |
| Leu | CUC | 239 | 0.49 |  | Tyr | UAC | 259 | 0.49 | trnY-GUA | |
| Leu | CUG | 220 | 0.45 |  | Tyr | UAU | 791 | 1.51 |  | |
| Met | AUG | 629 | 1.00 | trnM-CAU | TER | UAA | 201 | 1.15 |  | |
| Asn | AAU | 919 | 1.50 | trnN-GUU | TER | UAG | 1.64 | 0.94 |  | |
| Asn | AAC | 307 | 0. 50 |  | TER | UGA | 161 | 0.92 |  | |

RCSU, relative synonymous codon usage.

**Table S4.** Substitution and evaluation rates in the plastid genes of *Quercus* compared to gymnosperms (standard deviation).

| **Categories** | ***Dn*** | | ***Ds*** | | ***Dn/Ds*** | |
| --- | --- | --- | --- | --- | --- | --- |
|  | *Quercus* | gymnosperms | *Quercus* | gymnosperms | *Quercus* | gymnosperms |
| Photosynthetic metabolism | 0.0027 (0.0023) | 0.0991 (0.7836) | 0.0412 (0.0281) | 0.8770 (0.3237) | 0.0674 (0.0214) | 0.2057 (0.0511) |
| Photosynthetic apparatus | 0.1496 (0.5687) | 0.0956 (0.1087) | 0.2403 (0.7891) | 0.6194 (0.0093) | 0.3302 (0.4131) | 0.2050 (0.1664) |
| Gene expression | 0.2095 (0.6143) | 0.1708 (0.0575) | 0.3108 (0.9067) | 1.0886 (0.9242) | 0.4684 (0.4048) | 0.3126 (0.0857) |
| Other | 0.4978 (1.1837) | 0.2903 (0.1033) | 0.6953 (1.6252) | 0.8657 (0.1747) | 0.5454 (0.2334) | 0.4036 (0.0511) |
| Average | 0.1927 (0.6384) | 0.1359 (0.1036) | 0.2936(0.8988) | 0.8293 (0.6128) | 0.3793 (0.3747) | 0.2568 (0.1489) |
| Z | -7.317 | | -8.080 | | -0.729 | |
| P | 0.000 | | 0.000 | | 0.466 | |


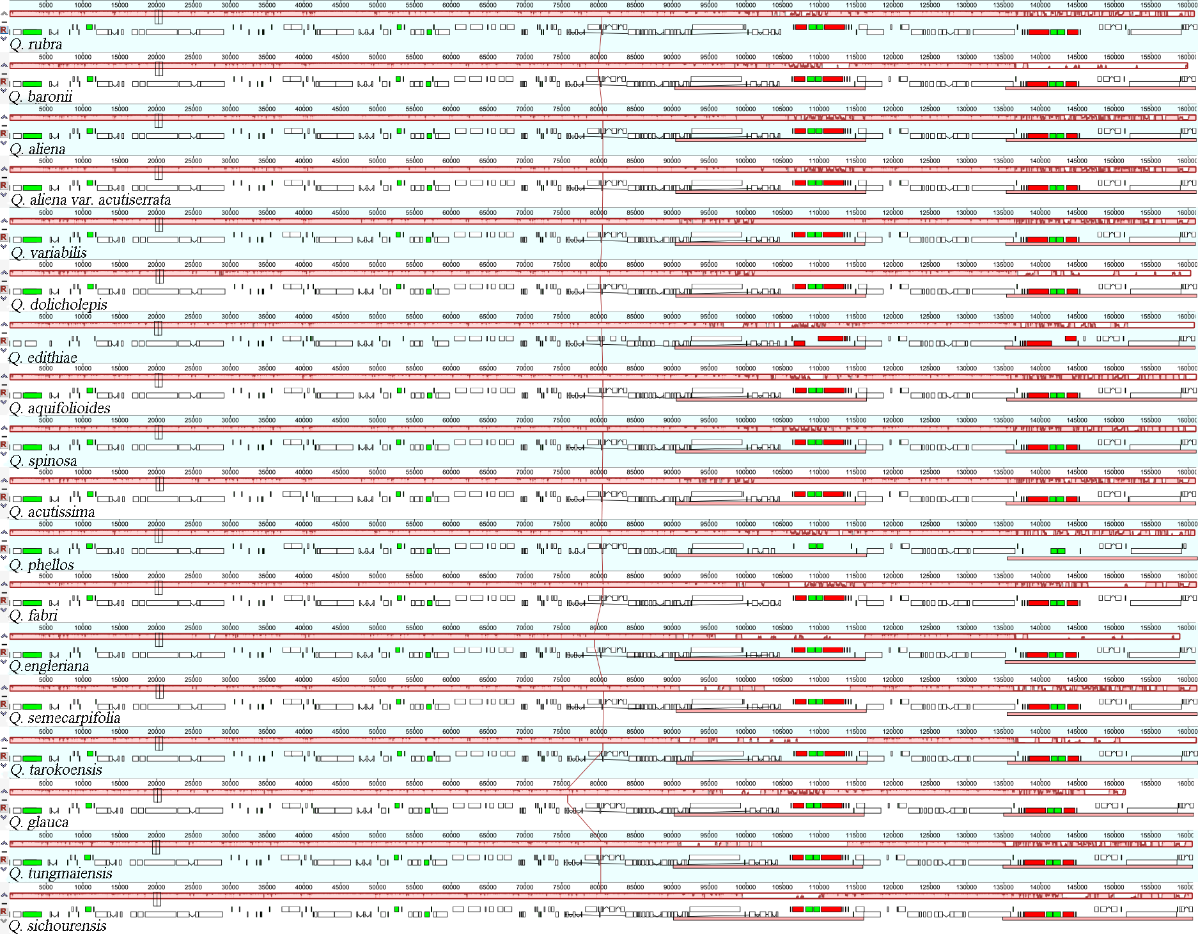


Figure S1. MAUVE alignments of the 18 *Quercus* plastomes, with *Q. aliena* set as the reference genome.


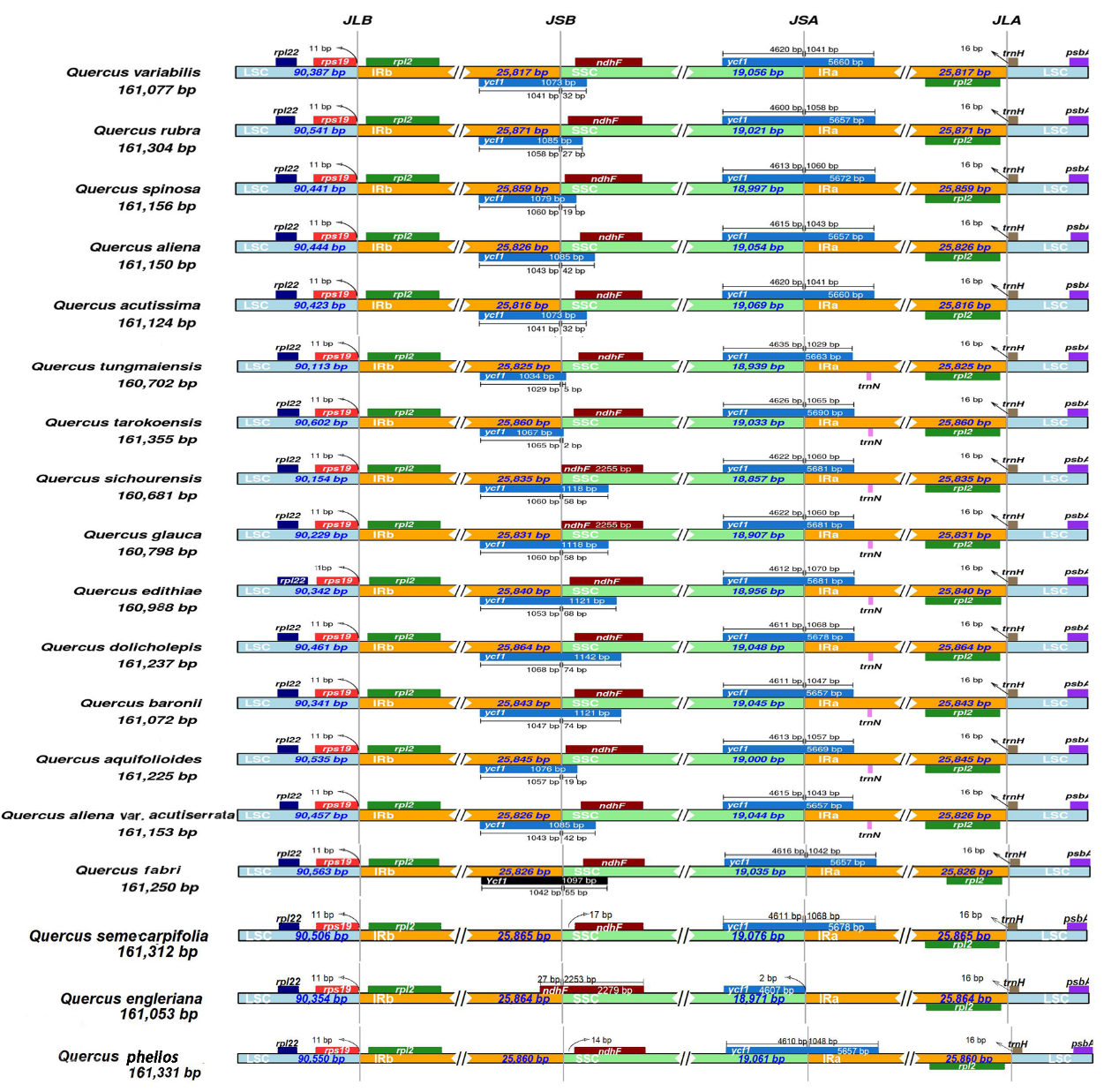


Figure S2. Comparisons of the LSC, SSC and IR regions among the plastomes of 18 *Quercus* species.


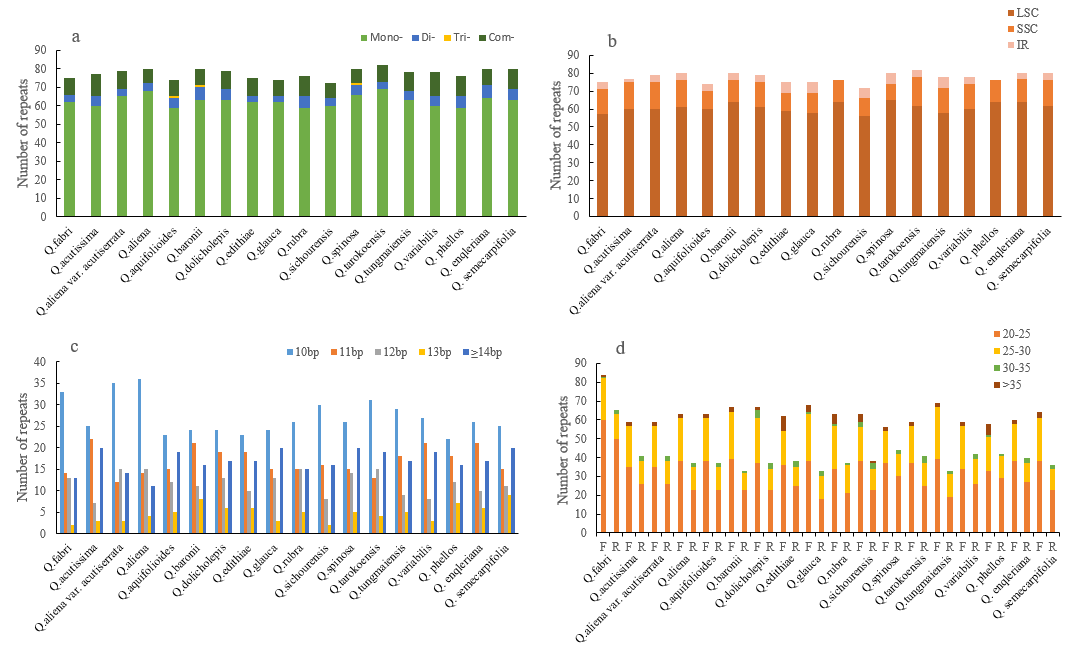


Figure S3. The distribution, type and presence of simple sequence repeats (SSRs) and long repeats in the plastomes of *Quercus*. a. Numbers of different SSR types; b. Number of SSRs in the LSC, SSC, and IR regions; c. Numbers of SSRs of each different length; d. Numbers of different long-repeat lengths per species.


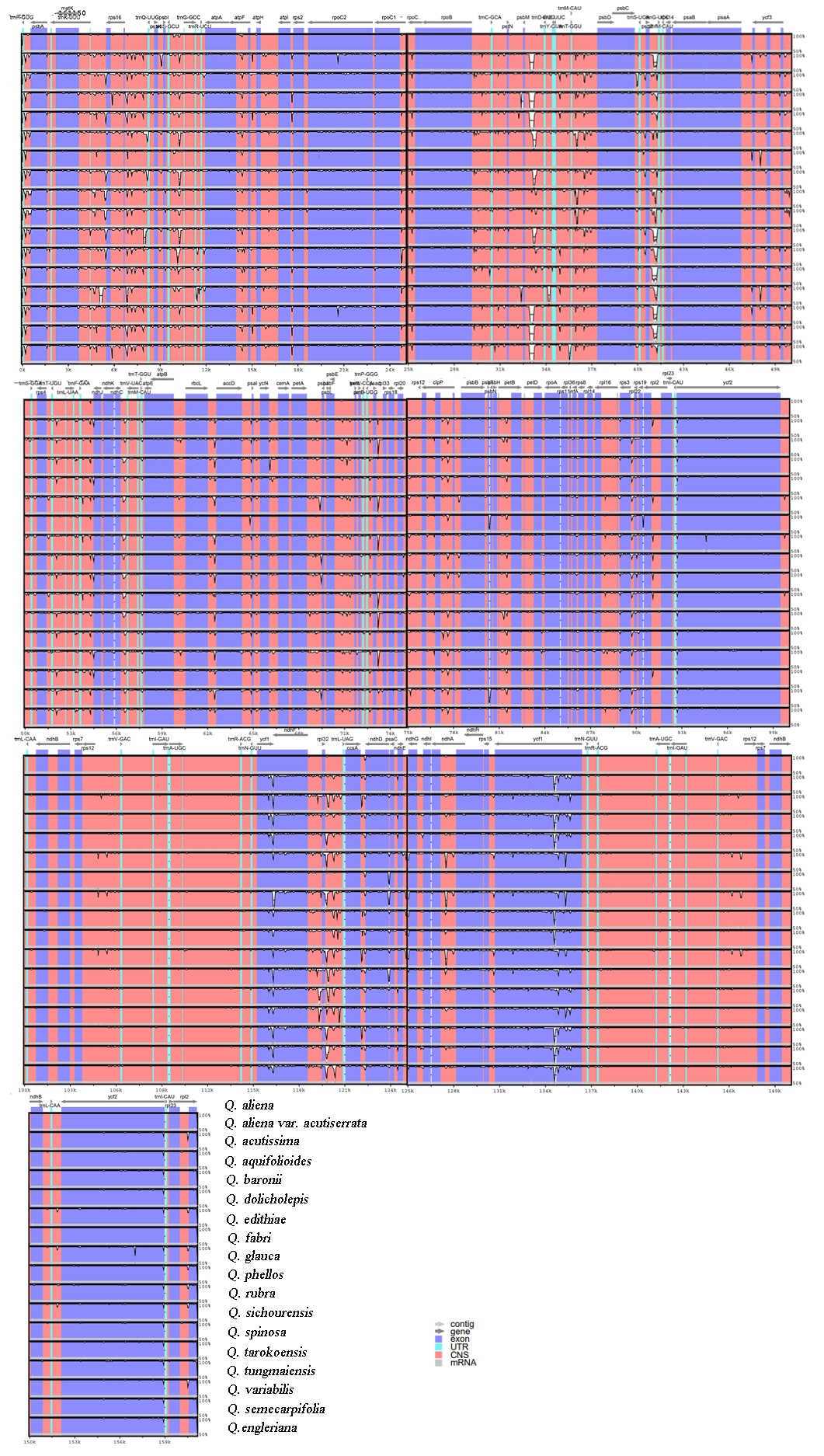


Figure S4. A comparison of the 18 plastomes using the mVISTA program. The red regions represent intergenic spacer regions, the blue regions represent genic regions and the white peaks represent differences in genomes. The y-axis represents the percentage identity (shown: 50-100%).
